# Supplementary material for: Comparative genomics analysis of three conserved plasmid families in the Western Hemisphere soft tick-borne relapsing fever borreliae provides insight into variation in genome structure and antigenic variation systems
Source: Microbiol Spectr. 2023 Sep 22;11(5):e00895-23. doi: 10.1128/spectrum.00895-23 (PMC10580987; doi:10.1128/spectrum.00895-23)
Supplement: Supplemental figures — Fig. S1 to S22. [file spectrum.00895-23-s0001.pdf]

Supplemental Figures

Figure S1

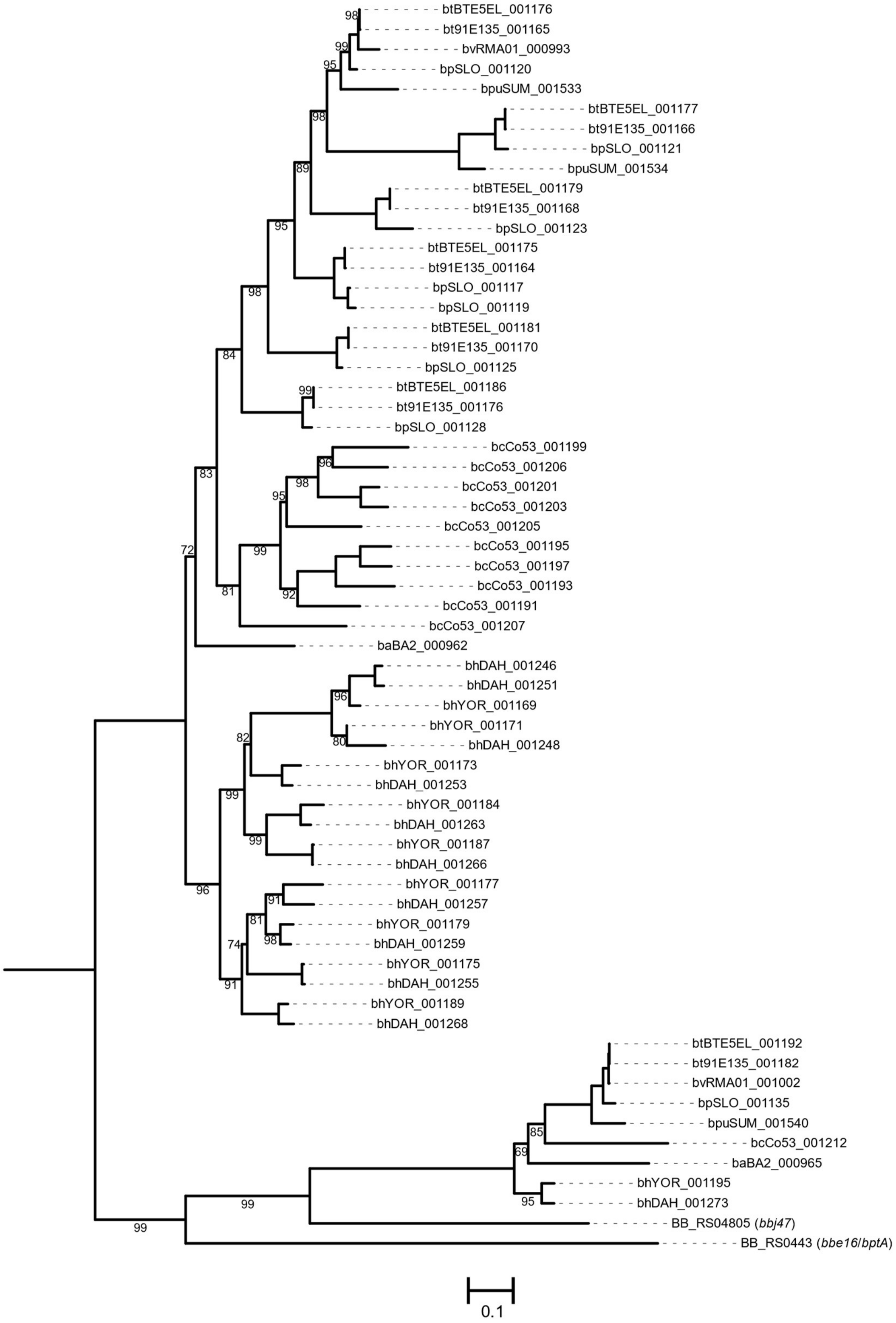

Figure S1. Phylogenetic analysis of genes related to *bptA*. A maximum likelihood tree was inferred using the nucleotide sequences of genes similar to *bptA* in *Borreliella* (*Borrelia*) *burgdorferi*. A midpoint rooted tree is shown. . Only branch supports less than 100% are shown. Branch supports indicated are the percentage of 1,000 ultrafast bootstrap replicates. The scale bar represents substitutions per site.

Figure S2

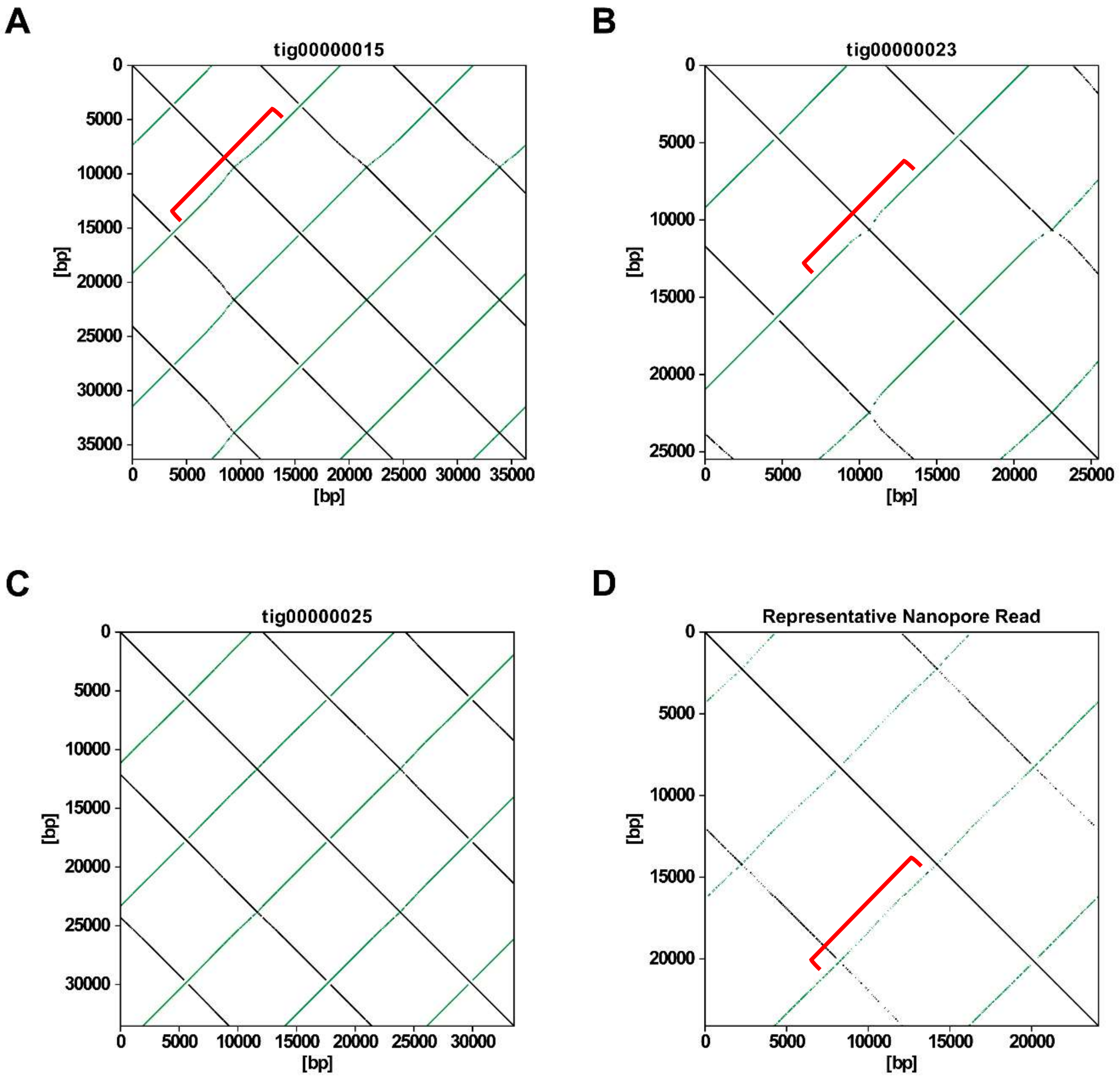

Figure S2. F27 plasmid family topology and size analysis. Self-dot plots are shown from the initial *B. hermsii* DAH assembly (**A**-**D**). Three different concatemeric contigs were generated in the initial assembly that depicted complete and incomplete inverted duplications (**A**-**C**). Dot plot analysis of a representative ONT read (~24kb) is shown in **D**. Dot plots were generated using FlexiDot. Black from top left to bottom right indicates direct sequence homology and green from bottom left to top right indicate inverted sequence homology. “Wavey” regions indicating areas of translocation issues through the nanopore due to inverted duplications are indicated by red brackets in the single read in **D** and are magnified in the contigs in **A** and **B**.

Figure S3

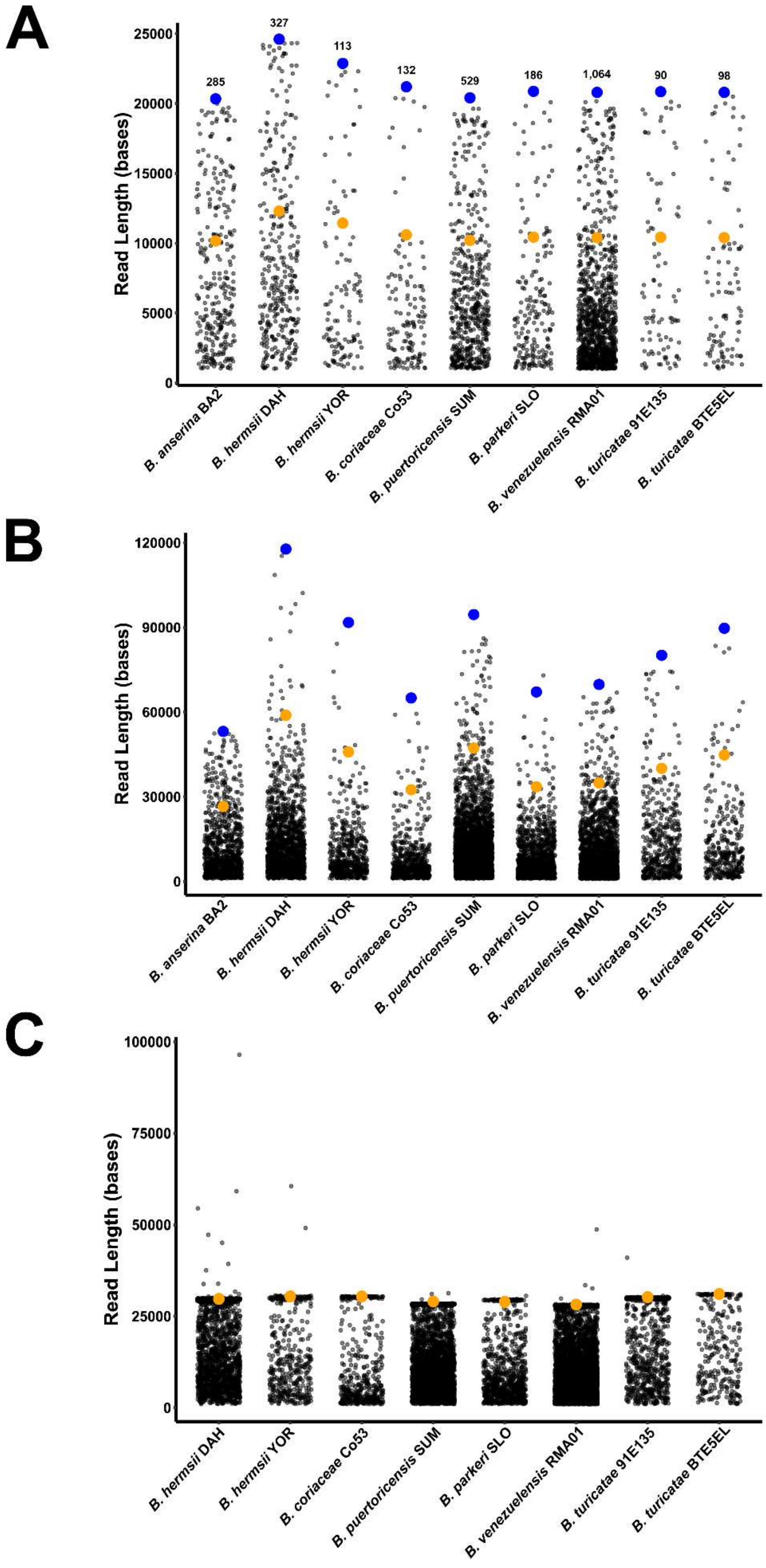
A
Read Length (bases)
25000
20000
15000
10000
5000
0
285
327
113
132
529
186
1,064
90
98
B. anserina BA2
B. hermsii DAH
B. hermsii YOR
B. coriaceae Co53
B. puertoricensis SUM
B. parkeri SLO
B. venezuelensis RMA01
B. turicatae 91E135
B. turicatae BTE5EL
B
Read Length (bases)
120000
90000
60000
30000
0
B. anserina BA2
B. hermsii DAH
B. hermsii YOR
B. coriaceae Co53
B. puertoricensis SUM
B. parkeri SLO
B. venezuelensis RMA01
B. turicatae 91E135
B. turicatae BTE5EL
C
Read Length (bases)
100000
75000
50000
25000
0
B. hermsii DAH
B. hermsii YOR
B. coriaceae Co53
B. puertoricensis SUM
B. parkeri SLO
B. venezuelensis RMA01
B. turicatae 91E135
B. turicatae BTE5EL

Figure S3. Read lengths of F27 plasmids and exemplar linear circular plasmids from the RBK dataset. The RBK dataset is the read dataset generated for the initial genome assembly of these isolates. **A** shows the read lengths of the primary reads mapping to the F27 plasmids in the RBK dataset. The orange dot indicates the assembled size of the F27 plasmid for that isolate and the blude dot indicates the length that is twice the size of the assembled plasmid. The numbers above each blue dot indicate the reads that were mapped to that plasmid and that are plotted. **B** shows the read lengths of the primary reads mapped to the F28 plasmid for the indicated isolates (see **Supplemental File 5** for RefSeq accessions and the plasmid identities). The F28 plasmids are linear plasmids, and these are shown as an example of what the read length distribution looks like for a linear plasmid from the RBK dataset. The orange dot indicates the assembled plasmid size, and the blue dot indicates the length twice the assembled size. C shows the read lengths of the primary reads mapped to representative circular plasmids for each isolate. The orange dot indicates the assembled plasmid size (see **Supplemental File 5** for RefSeq accessions and the plasmid identities).

Figure S4

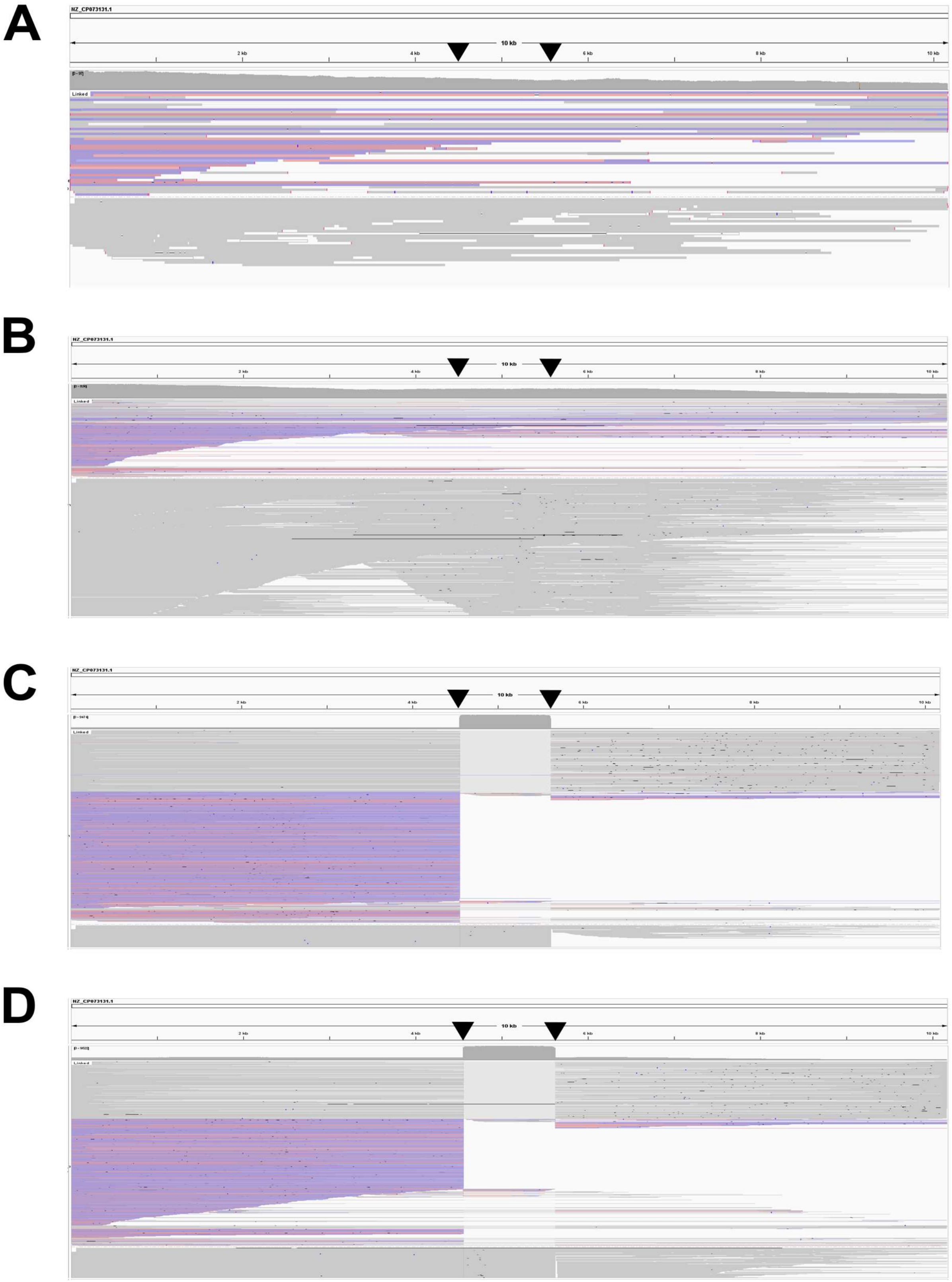
A
NZ_CP073131.1
10 kb
2 kb
4 kb
6 kb
8 kb
10 kb
Linked
B
NZ_CP073131.1
10 kb
Linked
C
NZ_CP073131.1
10 kb
Linked
D
NZ_CP073131.1
10 kb
Linked

Figure S4. Primary read mappings for digestion sequencing experiments of *B. anserina* BA2's F27 plasmid, lp10, NZ_CP073131.1 visualized in IGV. An IGV snapshot of read mappings are shown to demonstrate that fragment read length observed in **Figure 3** corresponds to the proper sequence for the predicted digestion fragment. Read coverage is indicated by a histogram at the top. The 'linked' indicates reads with supplemental mappings (using the 'link supplementary alignments' option). These are reads that would have been sequenced through the telomere (fragment C) so they would have a read mapping to both sides of the inverted repeats. Because the F27 plasmid is repetitive, and a read could map to either side the assignment is random so reads will look like a split read (grey with a line connecting them to either side of the B fragment) or they will map to the same side (overlapping blue and red to indicate an inverted repeat). Below the linked reads, read mapping is indicated by gray lines. The B fragment is located between the black triangles indicating where the restriction enzyme would cleave. **A** shows read mappings from the untreated condition. **B** shows read mappings from the S1 nuclease digestion. **C** shows read mappings from the restriction enzyme only digestion. **D** shows read mappings from the S1 nuclease and restriction enzyme double digestion. Where reads conflict in sequence with the reference, the conflicting base in the read is colored green for adenine, blue for cytosine, red for thymine, and yellow for guanine. Gaps or deletions are shown in the read as a black line. Insertions are indicated with a purple line in the read; however, indels <10bp have been masked.

Figure S5

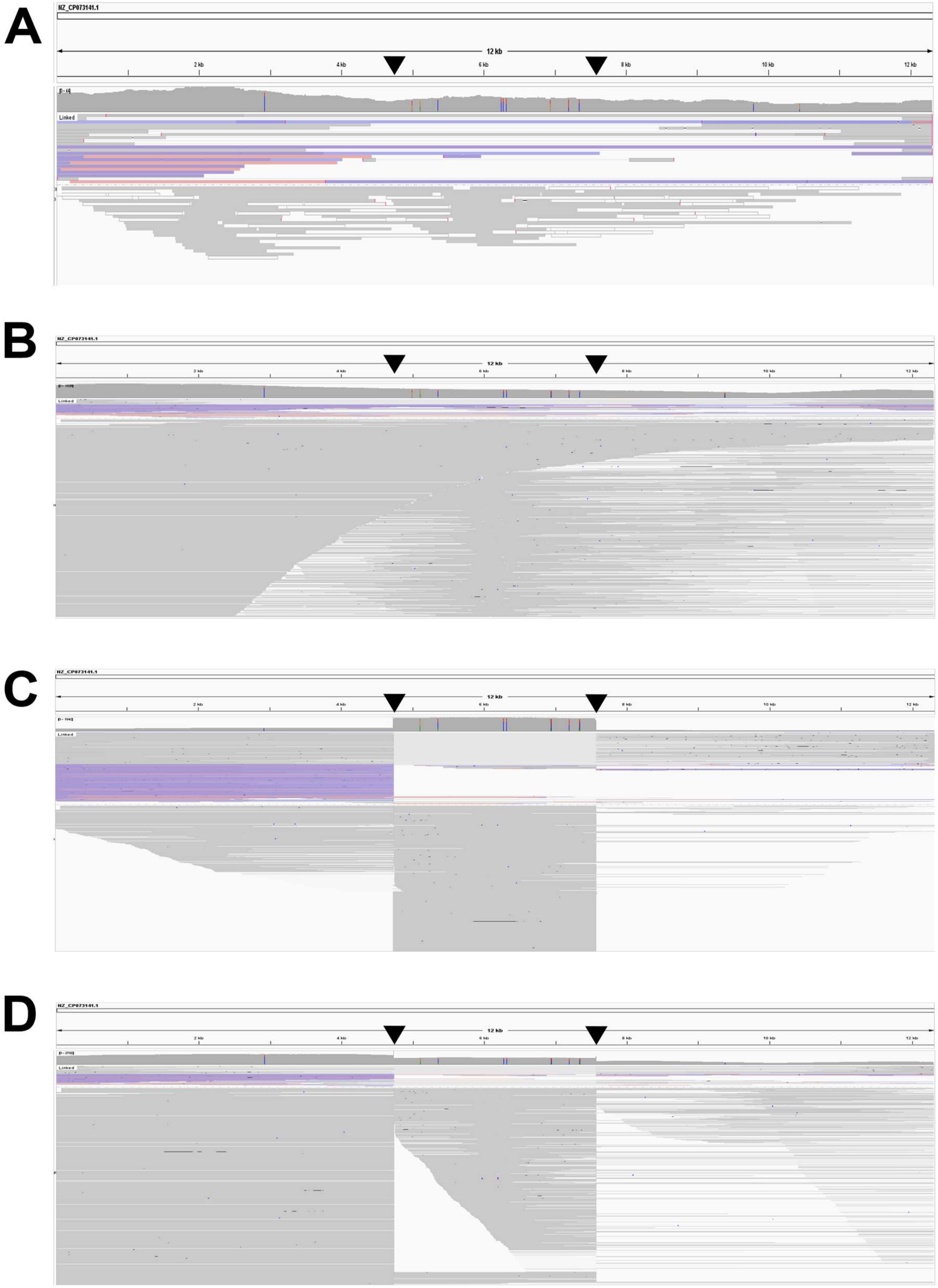

Figure S5. Primary read mappings for digestion sequencing experiments of *B. hermsii* DAH's F27 plasmid, lp12, NZ_CP073141.1 visualized in IGV. An IGV snapshot of read mappings are shown to demonstrate that fragment read length observed in **Figure 3** corresponds to the proper sequence for the predicted digestion fragment. Read coverage is indicated by a histogram at the top. The 'linked' indicates reads with supplemental mappings (using the 'link supplementary alignments' option). These are reads that would have been sequenced through the telomere (fragment C) so they would have a read mapping to both sides of the inverted repeats. Because the F27 plasmid is repetitive, and a read could map to either side the assignment is random so reads will look like a split read (grey with a line connecting them to either side of the B fragment) or they will map to the same side (overlapping blue and red to indicate an inverted repeat). Below the linked reads, read mapping is indicated by gray lines. The B fragment is located between the black triangles indicating where the restriction enzyme would cleave. **A** shows read mappings from the untreated condition. **B** shows read mappings from the S1 nuclease digestion. **C** shows read mappings from the restriction enzyme only digestion. **D** shows read mappings from the S1 nuclease and restriction enzyme double digestion. Where reads conflict in sequence with the reference, the conflicting base in the read is colored green for adenine, blue for cytosine, red for thymine, and yellow for guanine. Gaps or deletions are shown in the read as a black line. Insertions are indicated with a purple line in the read; however, indels <10bp have been masked.

Figure S6

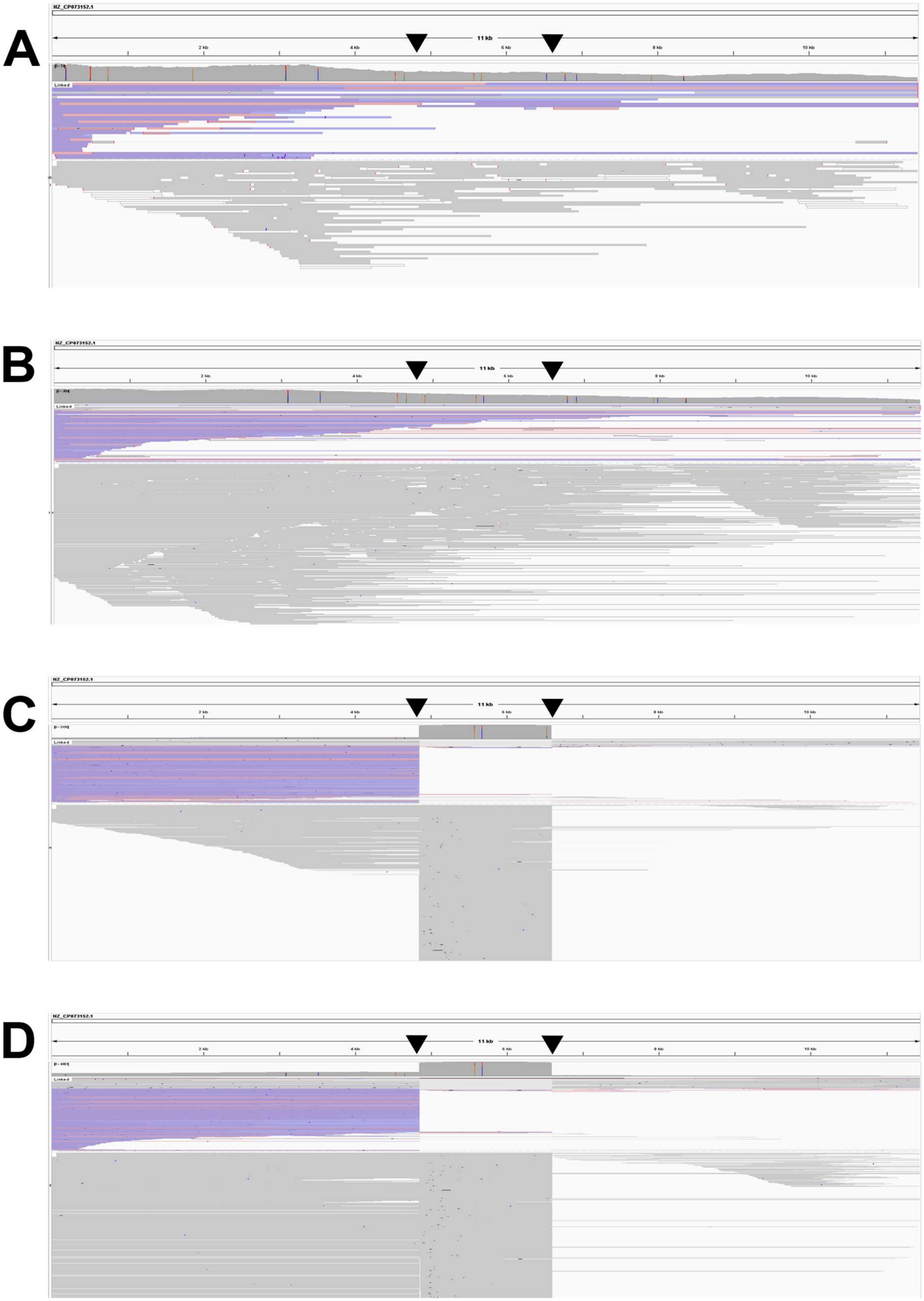

Figure S6. Primary read mappings for digestion sequencing experiments of *B. hermsii* YOR's F27 plasmid, lp11, NZ_CP073152.1 visualized in IGV. An IGV snapshot of read mappings are shown to demonstrate that fragment read length observed in **Figure 3** corresponds to the proper sequence for the predicted digestion fragment. Read coverage is indicated by a histogram at the top. The 'linked' indicates reads with supplemental mappings (using the 'link supplementary alignments' option). These are reads that would have been sequenced through the telomere (fragment C) so they would have a read mapping to both sides of the inverted repeats. Because the F27 plasmid is repetitive, and a read could map to either side the assignment is random so reads will look like a split read (grey with a line connecting them to either side of the B fragment) or they will map to the same side (overlapping blue and red to indicate an inverted repeat). Below the linked reads, read mapping is indicated by gray lines. The B fragment is located between the black triangles indicating where the restriction enzyme would cleave. **A** shows read mappings from the untreated condition. **B** shows read mappings from the S1 nuclease digestion. **C** shows read mappings from the restriction enzyme only digestion. **D** shows read mappings from the S1 nuclease and restriction enzyme double digestion. Where reads conflict in sequence with the reference, the conflicting base in the read is colored green for adenine, blue for cytosine, red for thymine, and yellow for guanine. Gaps or deletions are shown in the read as a black line. Insertions are indicated with a purple line in the read; however, indels <10bp have been masked.

Figure S7

A

B

C

D

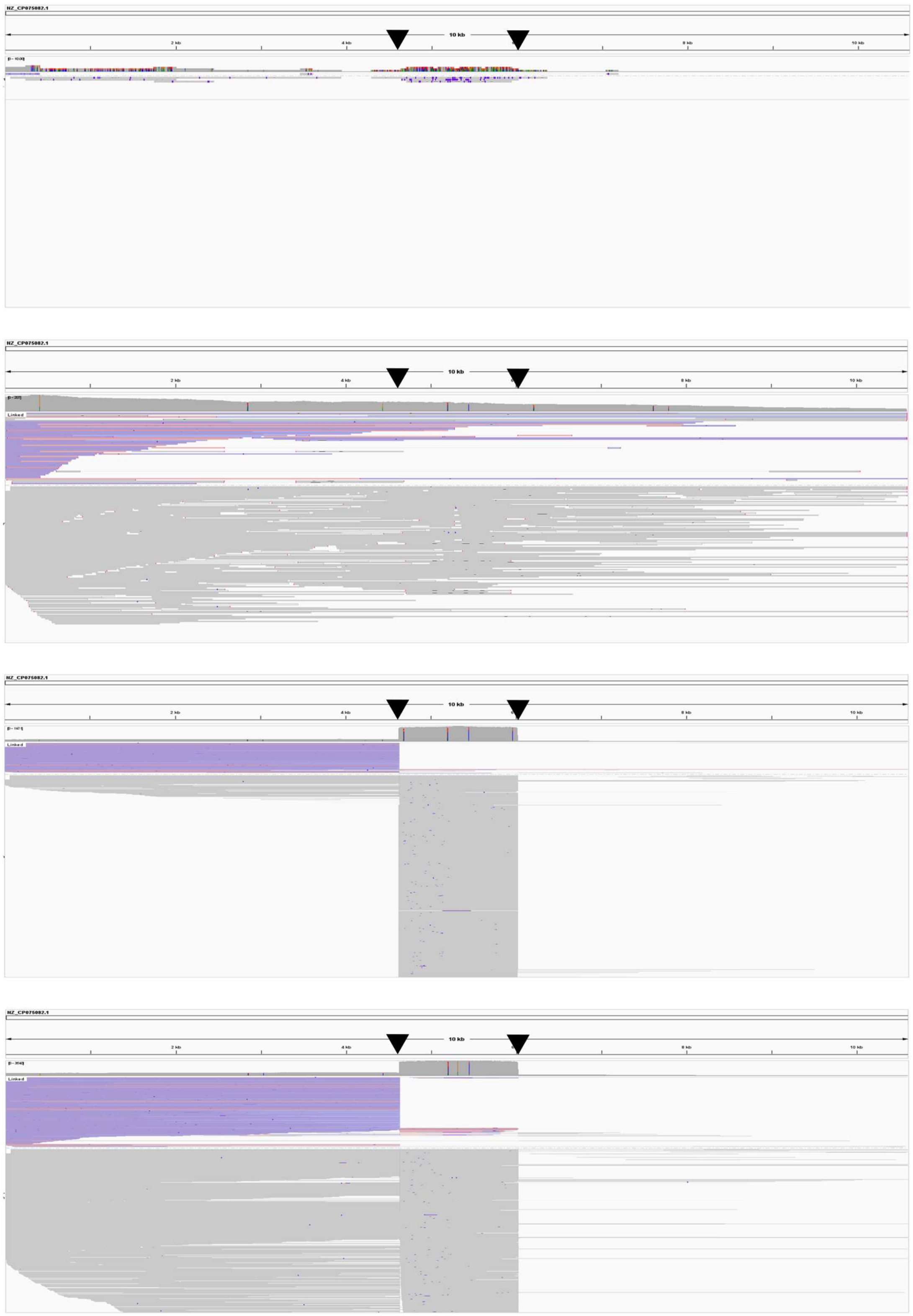

Figure S7. Primary read mappings for digestion sequencing experiments of *B. coriaceae* Co53's F27 plasmid, lp11, NZ_CP075082.1 visualized in IGV. An IGV snapshot of read mappings are shown to demonstrate that fragment read length observed in **Figure 3** corresponds to the proper sequence for the predicted digestion fragment. Read coverage is indicated by a histogram at the top. The 'linked' indicates reads with supplemental mappings (using the 'link supplementary alignments' option). These are reads that would have been sequenced through the telomere (fragment C) so they would have a read mapping to both sides of the inverted repeats. Because the F27 plasmid is repetitive, and a read could map to either side the assignment is random so reads will look like a split read (grey with a line connecting them to either side of the B fragment) or they will map to the same side (overlapping blue and red to indicate an inverted repeat). Below the linked reads, read mapping is indicated by gray lines. The B fragment is located between the black triangles indicating where the restriction enzyme would cleave. **A** shows read mappings from the untreated condition. **B** shows read mappings from the S1 nuclease digestion. **C** shows read mappings from the restriction enzyme only digestion. **D** shows read mappings from the S1 nuclease and restriction enzyme double digestion. Where reads conflict in sequence with the reference, the conflicting base in the read is colored green for adenine, blue for cytosine, red for thymine, and yellow for guanine. Gaps or deletions are shown in the read as a black line. Insertions are indicated with a purple line in the read; however, indels <10bp have been masked.

Figure S8

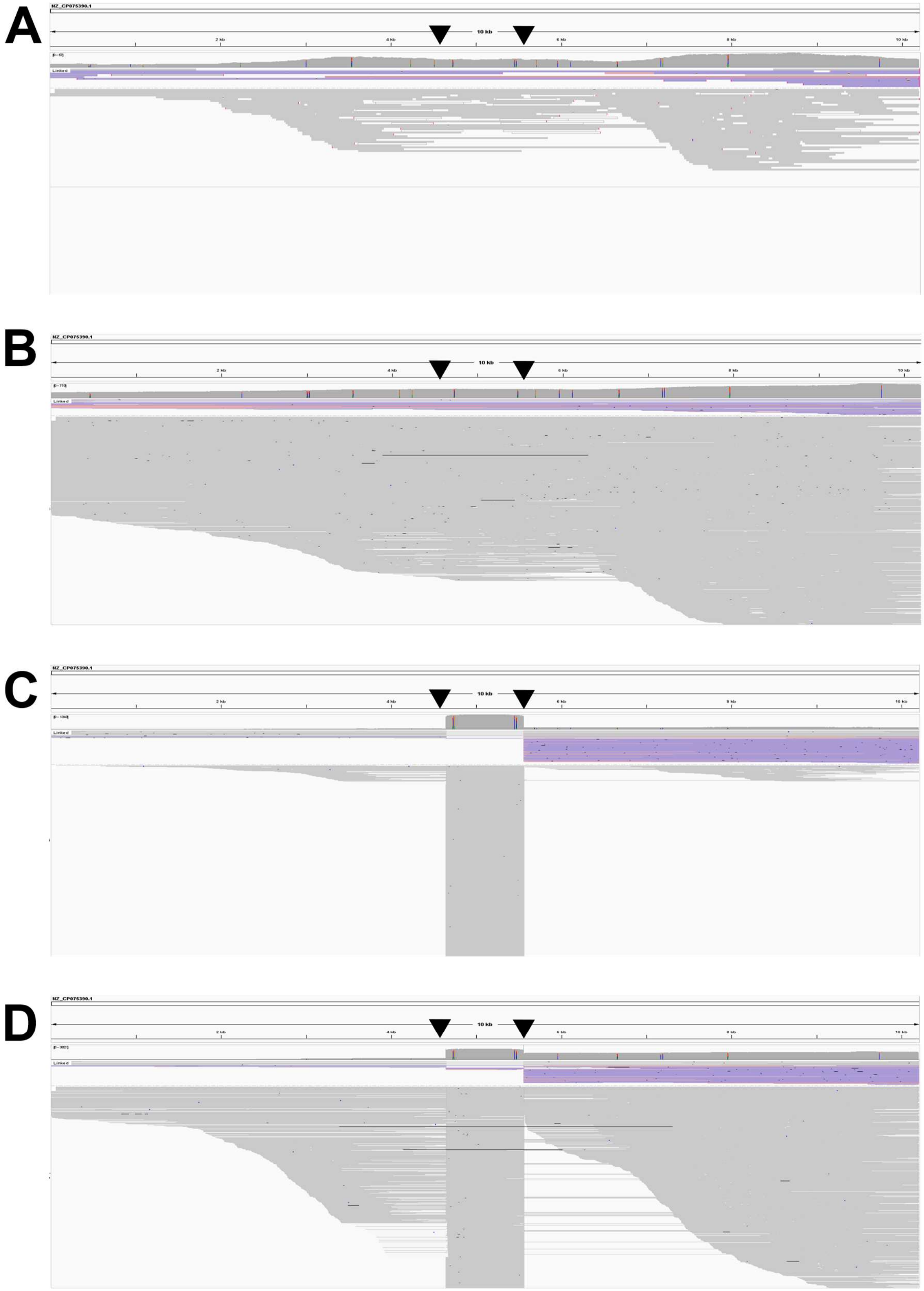

Figure S8. Primary read mappings for digestion sequencing experiments of *B. puertoricensis* SUM's F27 plasmid, lp10, NZ_CP075390.1 visualized in IGV. An IGV snapshot of read mappings are shown to demonstrate that fragment read length observed in **Figure 3** corresponds to the proper sequence for the predicted digestion fragment. Read coverage is indicated by a histogram at the top. The 'linked' indicates reads with supplemental mappings (using the 'link supplementary alignments' option). These are reads that would have been sequenced through the telomere (fragment C) so they would have a read mapping to both sides of the inverted repeats. Because the F27 plasmid is repetitive, and a read could map to either side, the assignment is random so reads will look like a split read (grey with a line connecting them to either side of the B fragment) or they will map to the same side (overlapping blue and red to indicate an inverted repeat). Below the linked reads, read mapping is indicated by gray lines. Below the linked reads, read mapping is indicated by gray lines. The B fragment is located between the black triangles indicating where the restriction enzyme would cleave. **A** shows read mappings from the untreated condition. **B** shows read mappings from the S1 nuclease digestion. **C** shows read mappings from the restriction enzyme only digestion. **D** shows read mappings from the S1 nuclease and restriction enzyme double digestion. Where reads conflict in sequence with the reference, the conflicting base in the read is colored green for adenine, blue for cytosine, red for thymine, and yellow for guanine. Gaps or deletions are shown in the read as a black line. Insertions are indicated with a purple line in the read; however, indels <10bp have been masked.

Figure S9

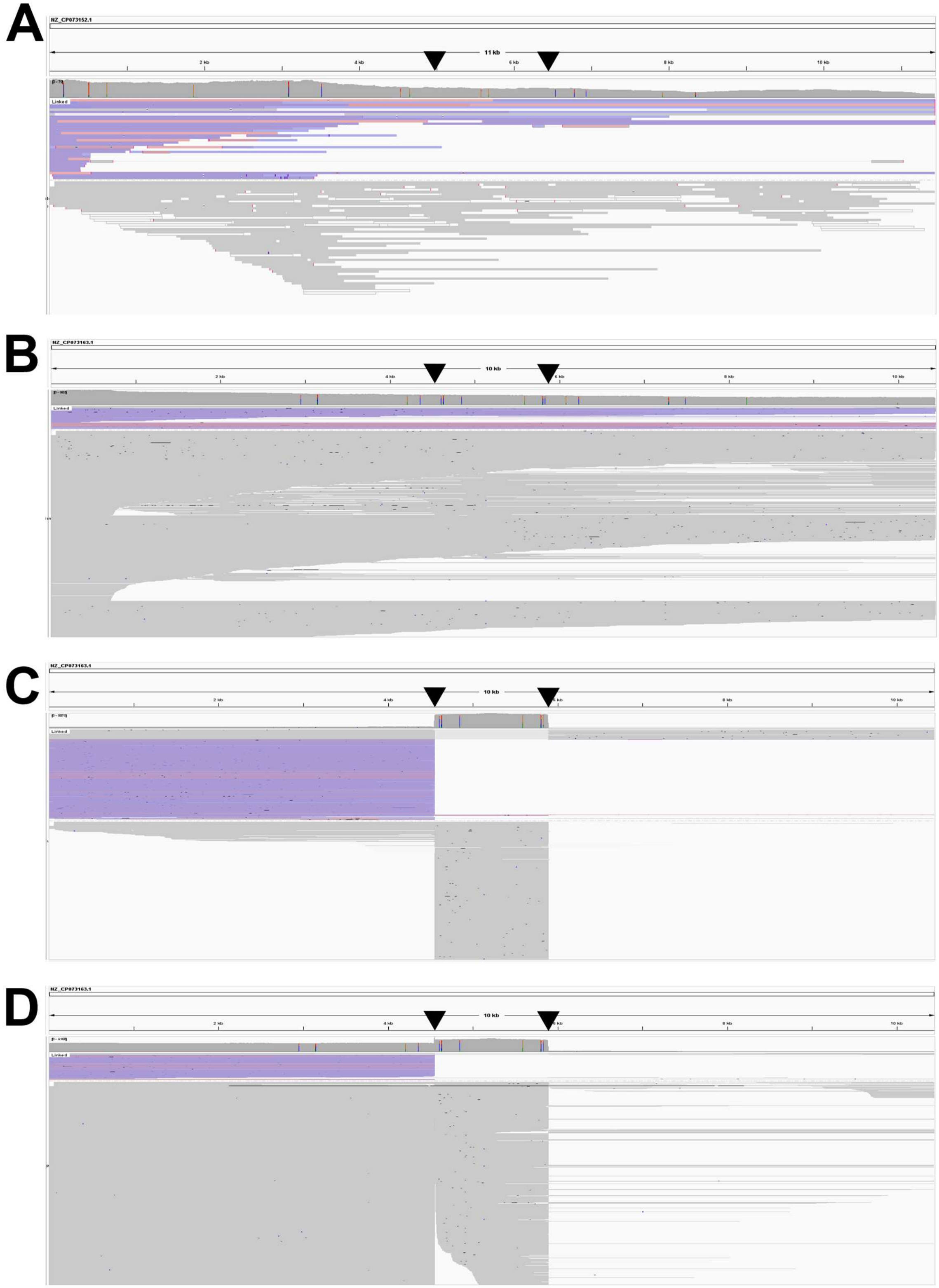

Figure S9. Primary read mappings for digestion sequencing experiments of *B. parkeri* SLO 's F27 plasmid, lp10, NZ_CP073163.1 visualized in IGV. An IGV snapshot of read mappings are shown to demonstrate that fragment read length observed in **Figure 3** corresponds to the proper sequence for the predicted digestion fragment. Read coverage is indicated by a histogram at the top. The 'linked' indicates reads with supplemental mappings (using the 'link supplementary alignments' option). These are reads that would have been sequenced through the telomere (fragment C) so they would have a read mapping to both sides of the inverted repeats. Because the F27 plasmid is repetitive, and a read could map to either side the assignment is random so reads will look like a split read (grey with a line connecting them to either side of the B fragment) or they will map to the same side (overlapping blue and red to indicate an inverted repeat). Below the linked reads, read mapping is indicated by gray lines. The B fragment is located between the black triangles indicating where the restriction enzyme would cleave. **A** shows read mappings from the untreated condition. **B** shows read mappings from the S1 nuclease digestion. **C** shows read mappings from the restriction enzyme only digestion. **D** shows read mappings from the S1 nuclease and restriction enzyme double digestion. Where reads conflict in sequence with the reference, the conflicting base in the read is colored green for adenine, blue for cytosine, red for thymine, and yellow for guanine. Gaps or deletions are shown in the read as a black line. Insertions are indicated with a purple line in the read; however, indels <10bp have been masked.

Figure S10

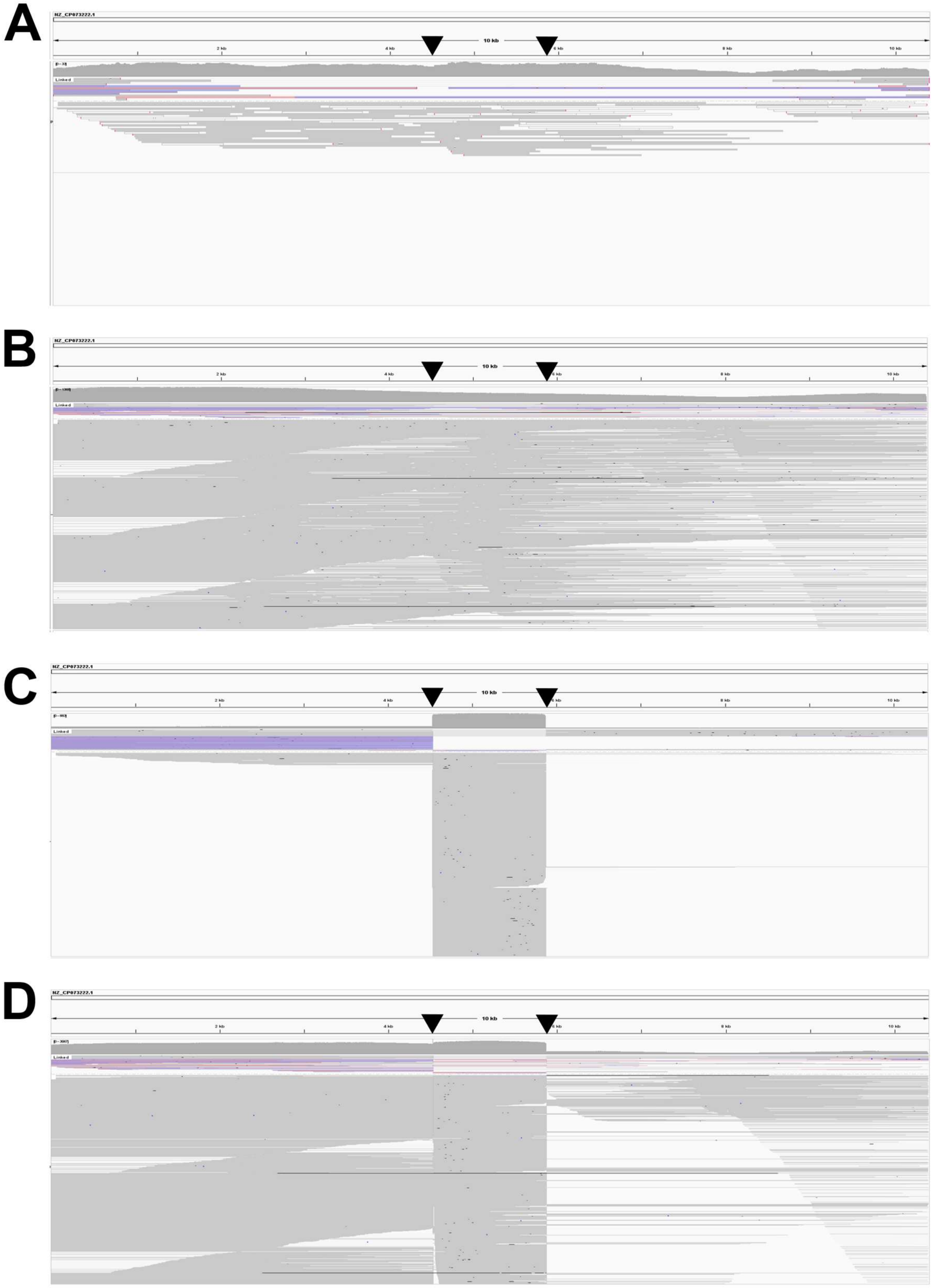

Figure S10. Primary read mappings for digestion sequencing experiments of *B. venezuelensis* RMA01's F27 plasmid, lp10, NZ_CP073222.1 visualized in IGV. An IGV snapshot of read mappings are shown to demonstrate that fragment read length observed in **Figure 3** corresponds to the proper sequence for the predicted digestion fragment. Read coverage is indicated by a histogram at the top. The 'linked' indicates reads with supplemental mappings (using the 'link supplementary alignments' option). These are reads that would have been sequenced through the telomere (fragment C) so they would have a read mapping to both sides of the inverted repeats. Because the F27 plasmid is repetitive, and a read could map to either side the assignment is random so reads will look like a split read (grey with a line connecting them to either side of the B fragment) or they will map to the same side (overlapping blue and red to indicate an inverted repeat). Below the linked reads, read mapping is indicated by gray lines. The B fragment is located between the black triangles indicating where the restriction enzyme would cleave. **A** shows read mappings from the untreated condition. **B** shows read mappings from the S1 nuclease digestion. **C** shows read mappings from the restriction enzyme only digestion. **D** shows read mappings from the S1 nuclease and restriction enzyme double digestion. Where reads conflict in sequence with the reference, the conflicting base in the read is colored green for adenine, blue for cytosine, red for thymine, and yellow for guanine. Gaps or deletions are shown in the read as a black line. Insertions are indicated with a purple line in the read; however, indels <10bp have been masked.

Figure S11

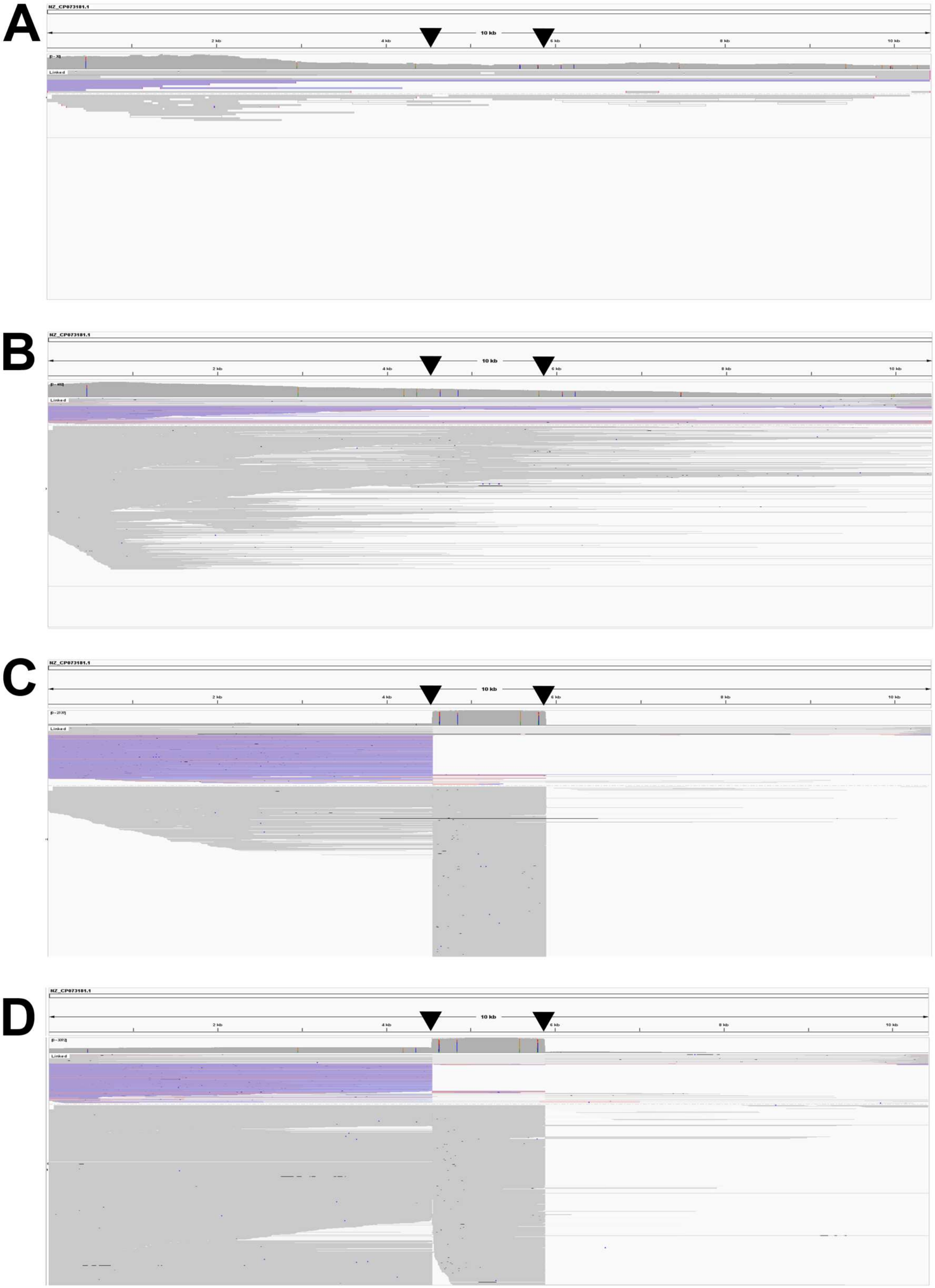

Figure S11. Primary read mappings for digestion sequencing experiments of *B. turicatae* 91E135's F27 plasmid, lp10, NZ_CP073181.1 visualized in IGV. An IGV snapshot of read mappings are shown to demonstrate that fragment read length observed in **Figure 3** corresponds to the proper sequence for the predicted digestion fragment. Read coverage is indicated by a histogram at the top. The 'linked' indicates reads with supplemental mappings (using the 'link supplementary alignments' option). These are reads that would have been sequenced through the telomere (fragment C) so they would have a read mapping to both sides of the inverted repeats. Because the F27 plasmid is repetitive, and a read could map to either side the assignment is random so reads will look like a split read (grey with a line connecting them to either side of the B fragment) or they will map to the same side (overlapping blue and red to indicate an inverted repeat). Below the linked reads, read mapping is indicated by gray lines. The B fragment is located between the black triangles indicating where the restriction enzyme would cleave. **A** shows read mappings from the untreated condition. **B** shows read mappings from the S1 nuclease digestion. **C** shows read mappings from the restriction enzyme only digestion. **D** shows read mappings from the S1 nuclease and restriction enzyme double digestion. Where reads conflict in sequence with the reference, the conflicting base in the read is colored green for adenine, blue for cytosine, red for thymine, and yellow for guanine. Gaps or deletions are shown in the read as a black line. Insertions are indicated with a purple line in the read; however, indels <10bp have been masked.

Figure S12

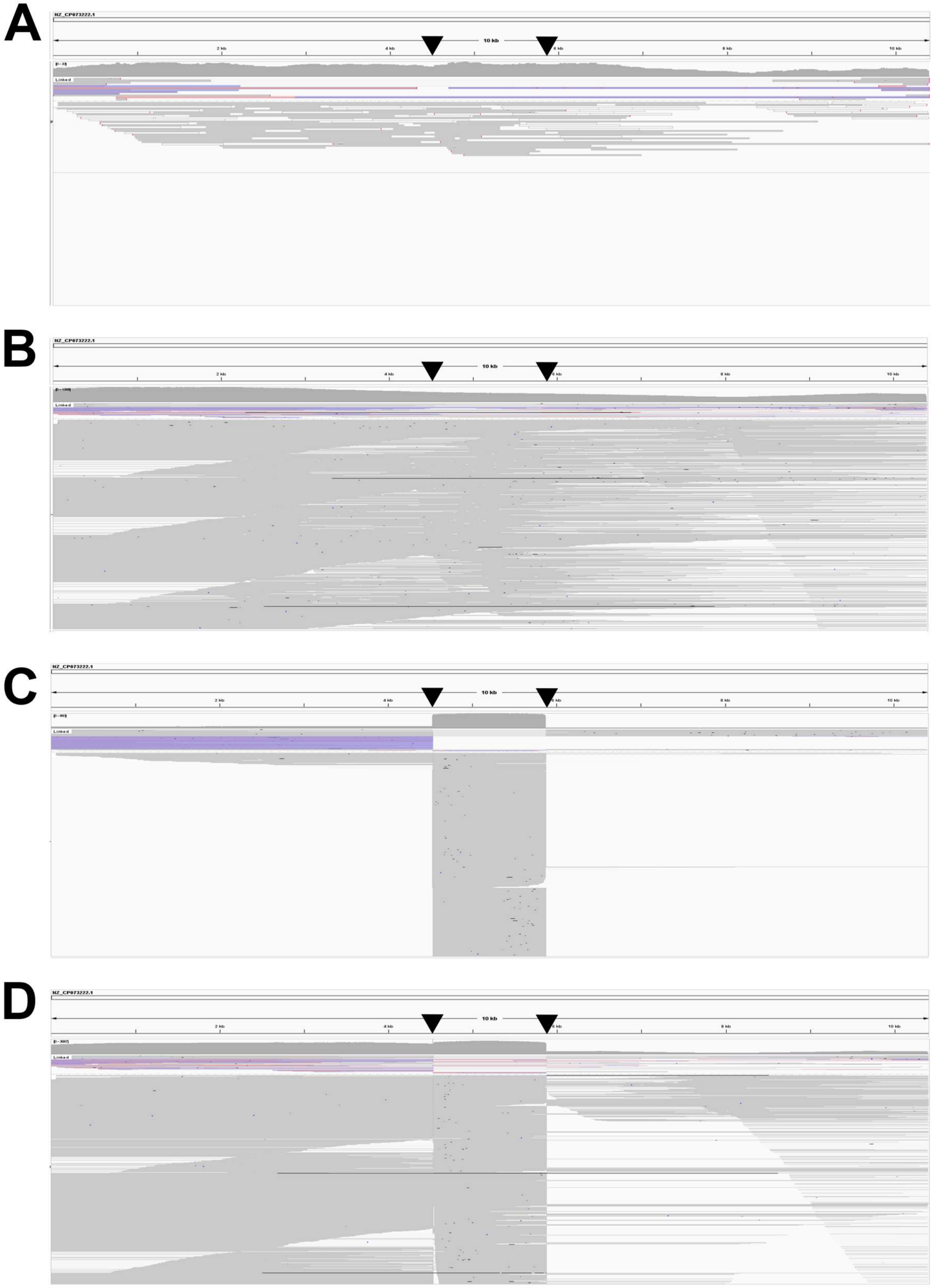

Figure S12. Primary read mappings for digestion sequencing experiments of *B. turicatae* BTE5EL's F27 plasmid, lp10, NZ_CP073197.1 visualized in IGV. An IGV snapshot of read mappings are shown to demonstrate that fragment read length observed in **Figure 3** corresponds to the proper sequence for the predicted digestion fragment. Read coverage is indicated by a histogram at the top. The 'linked' indicates reads with supplemental mappings (using the 'link supplementary alignments' option). These are reads that would have been sequenced through the telomere (fragment C) so they would have a read mapping to both sides of the inverted repeats. Because the F27 plasmid is repetitive, and a read could map to either side the assignment is random so reads will look like a split read (grey with a line connecting them to either side of the B fragment) or they will map to the same side (overlapping blue and red to indicate an inverted repeat). Below the linked reads, read mapping is indicated by gray lines. The B fragment is located between the black triangles indicating where the restriction enzyme would cleave. **A** shows read mappings from the untreated condition. **B** shows read mappings from the S1 nuclease digestion. **C** shows read mappings from the restriction enzyme only digestion. **D** shows read mappings from the S1 nuclease and restriction enzyme double digestion. Where reads conflict in sequence with the reference, the conflicting base in the read is colored green for adenine, blue for cytosine, red for thymine, and yellow for guanine. Gaps or deletions are shown in the read as a black line. Insertions are indicated with a purple line in the read; however, indels <10bp have been masked.

Figure S13

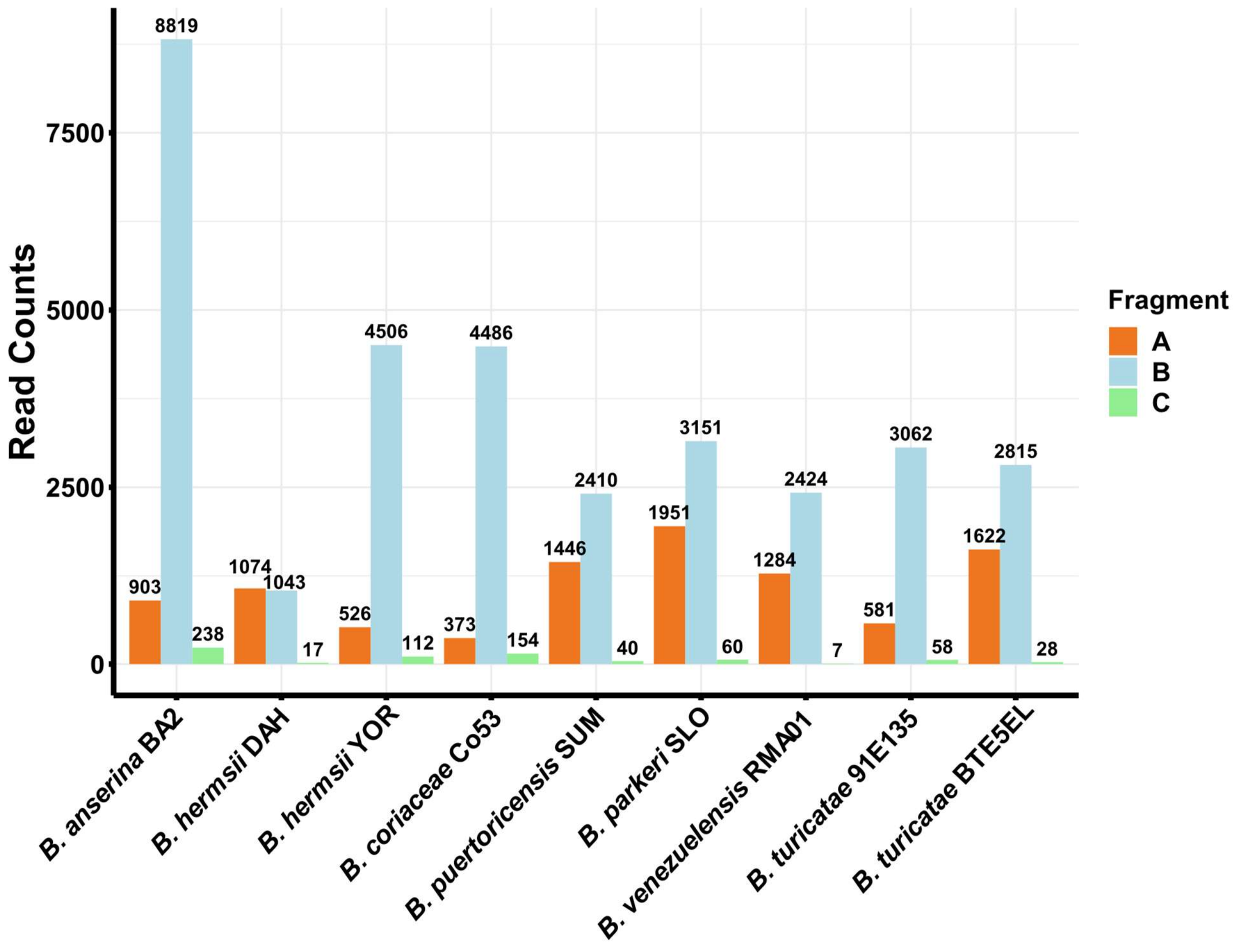

Figure S13. Read counts of plasmid digestion fragments. The read counts for the S1 nuclease and restriction enzyme double digestion fragments were graphed for each isolate. Read counts were generated by counting the reads with read lengths that fell between the fragment length minus 5% of overall fragment length and the fragment length plus 5% of the overall fragment length. The number above each bar indicates the number of reads detected for that fragment.

Figure S14

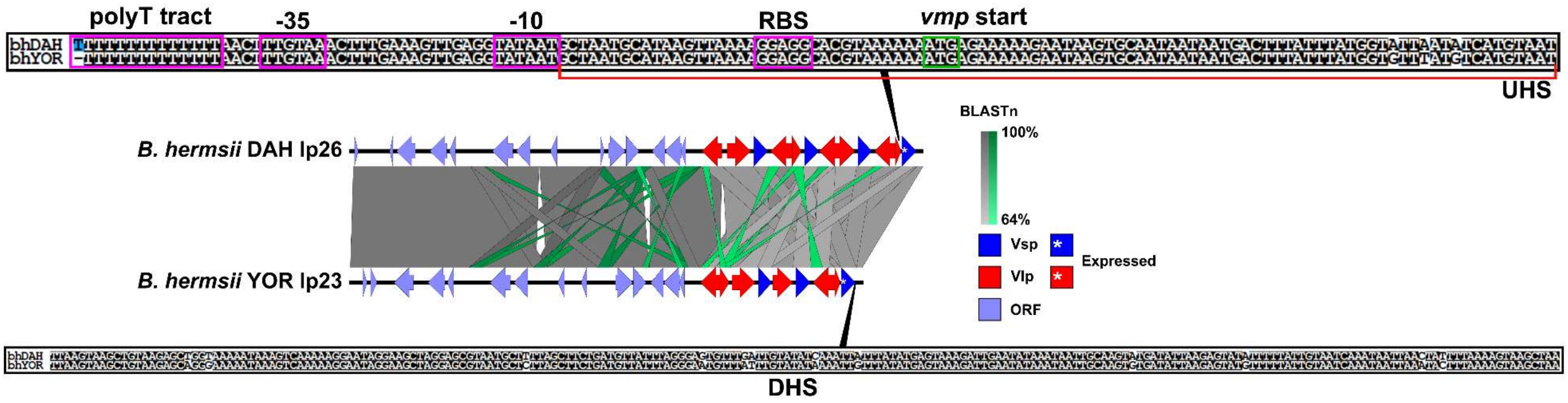

Figure S14. *Borrelia hermsii* antigenic variation Vmp expression plasmid alignment and visualization. The F20 plasmids of both *B. hermsii* isolates were aligned using EasyFig. The minimum BLASTn alignment was set to 250nt. BLASTn results scale from 64-100% going from light grey to dark grey whereas inversions scale the same but from light green to dark green. The *vmp* alleles and other open reading frames (ORFs) are shown in specific colors. The *vmp* in the expression site is indicated by an asterisk within the indicated ORF. The vmp promoter alignment is seen in the top sequence. Promoter features are annotated and boxed in purple (RBS=ribosome binding site). The ATG start codon is boxed in green. The upstream homology sequence (UHS) is indicated with a red bracket. The alignment of the downstream homology sequence (DHS) is seen below. For the alignments: a white text in a black square means the sequences are the same, black text in a white square indicates sequences are different, and white texts in blue squares indicates that that nucleotide matches the consensus for that position.

Figure S15

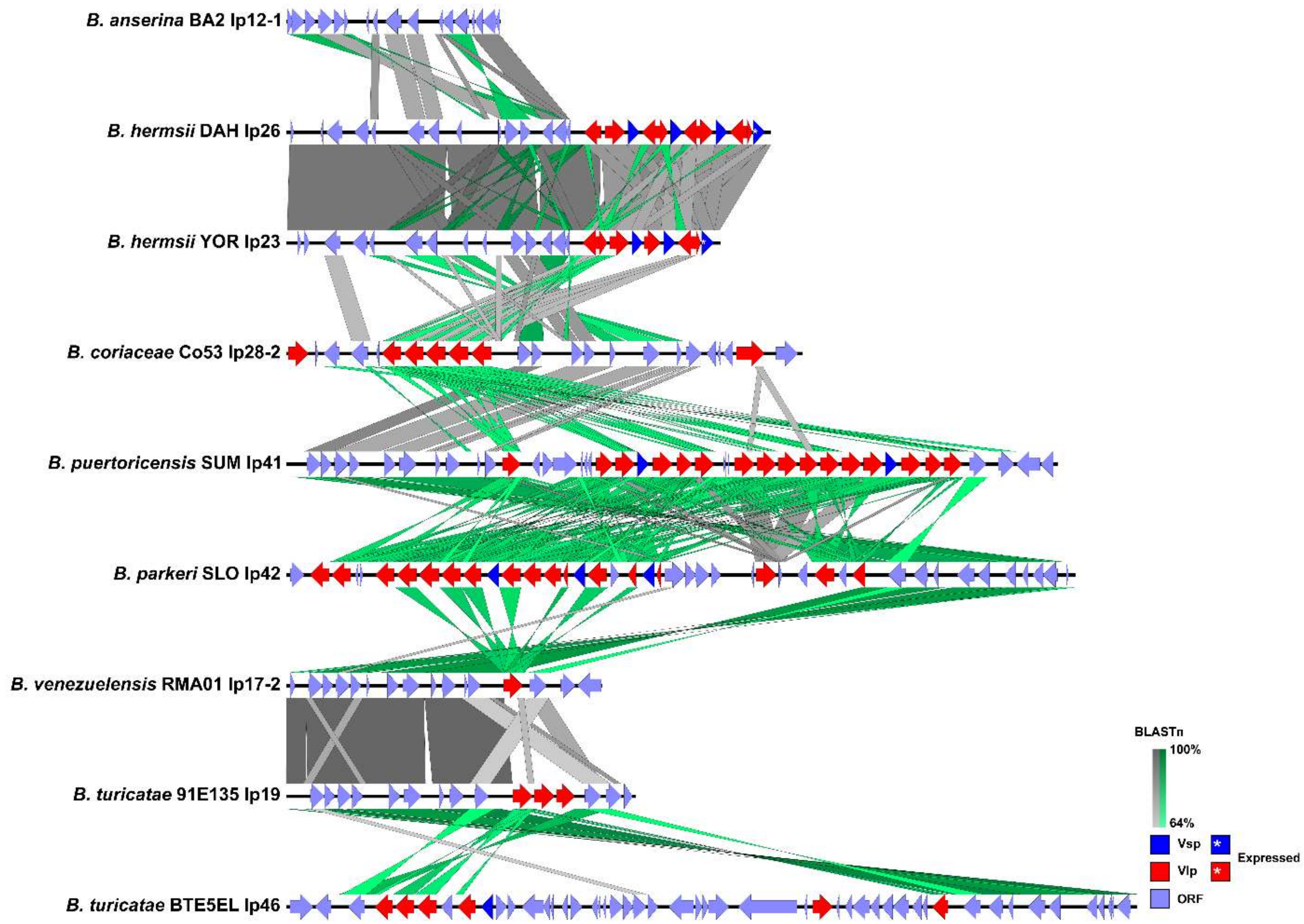

Figure S15. Alignment and visualization of the F20 plasmid family. All F20 plasmids were aligned using EasyFig demonstrating the lack of synteny across all species. The minimum BLASTn alignment was set to 250nt. BLASTn results scale from 64-100% going from light grey to dark grey whereas inversions scale the same but from light green to dark green. The *vmp* alleles and other open reading frames (ORFs) are shown in specific colors. The *vmp* in the expression site is indicated by an asterisk within the indicated ORF.

Figure S16

A

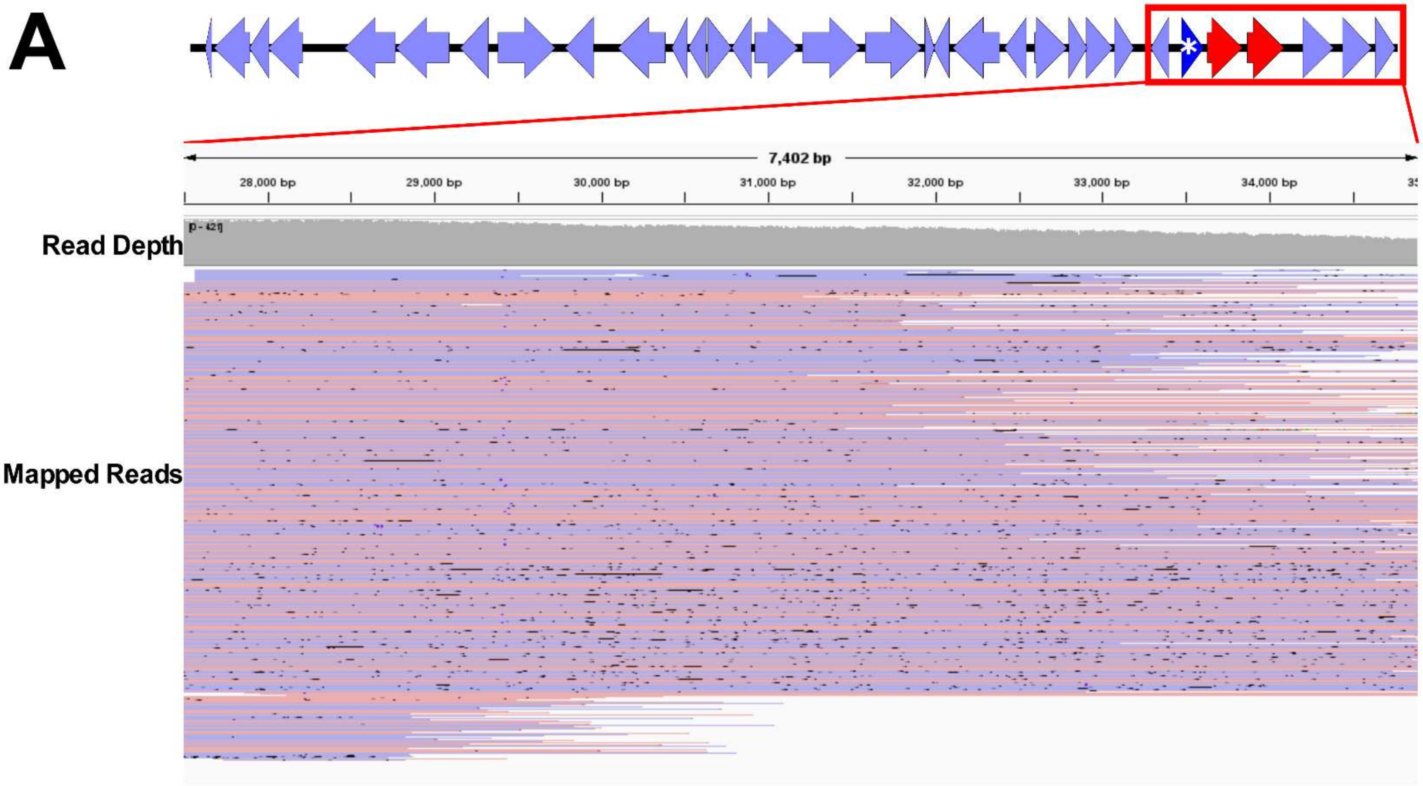

B

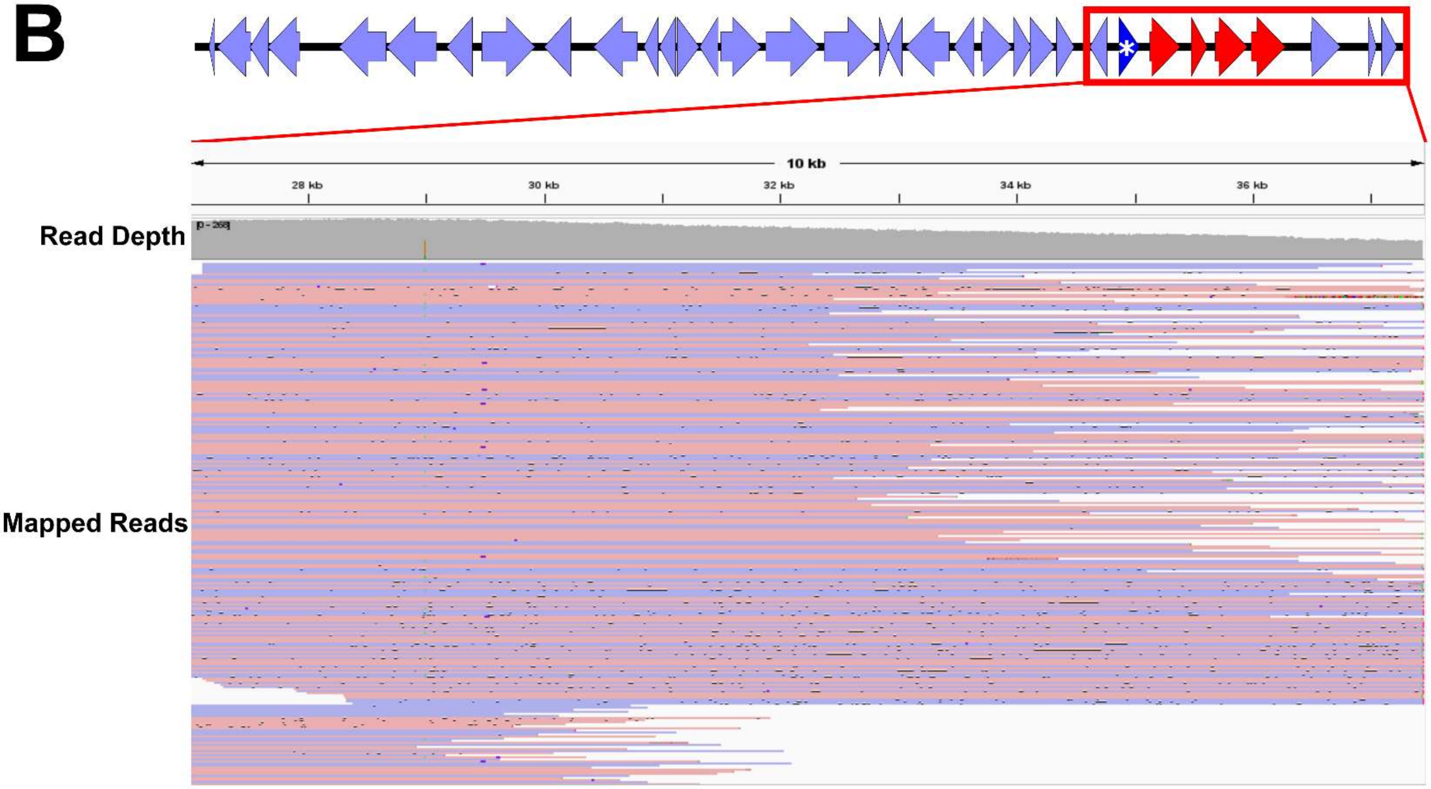

Figure S16. *B. venezuelensis* RMA01 F28 plasmid long-read mapping. Long-read mapping was performed on the lp35 (**A**) and lp37 (**B**) plasmids (F28 plasmids) of *B. venezuelensis* RMA01. Shown are the graphical representations of *B. venezuelensis* RMA01's F28 plasmids and the BAM file visualization in the Integrated Genomics Viewer of the highlighted 3' region starting from the conserved, upstream *oligopeptide permease-like protein* gene. In the graphical representation of the plasmids, the lavender arrows indicate ORFs and their directions, blue triangles indicate *vsp* alleles, and red indicates *vlp* alleles. The blue triangles with the asterisk indicate the *vmp* expression site. Supplementary alignments have been linked with the forward alignment shown in red with the reverse alignment overlayed in blue. Where reads conflict in sequence with the reference, the conflicting base in the read is colored green for adenine, blue for cytosine, red for thymine, and yellow for guanine. Gaps or deletions are shown in the read as a black line. Insertions are indicated with a purple line in the read; however, indels <10bp have been masked.

Figure S17

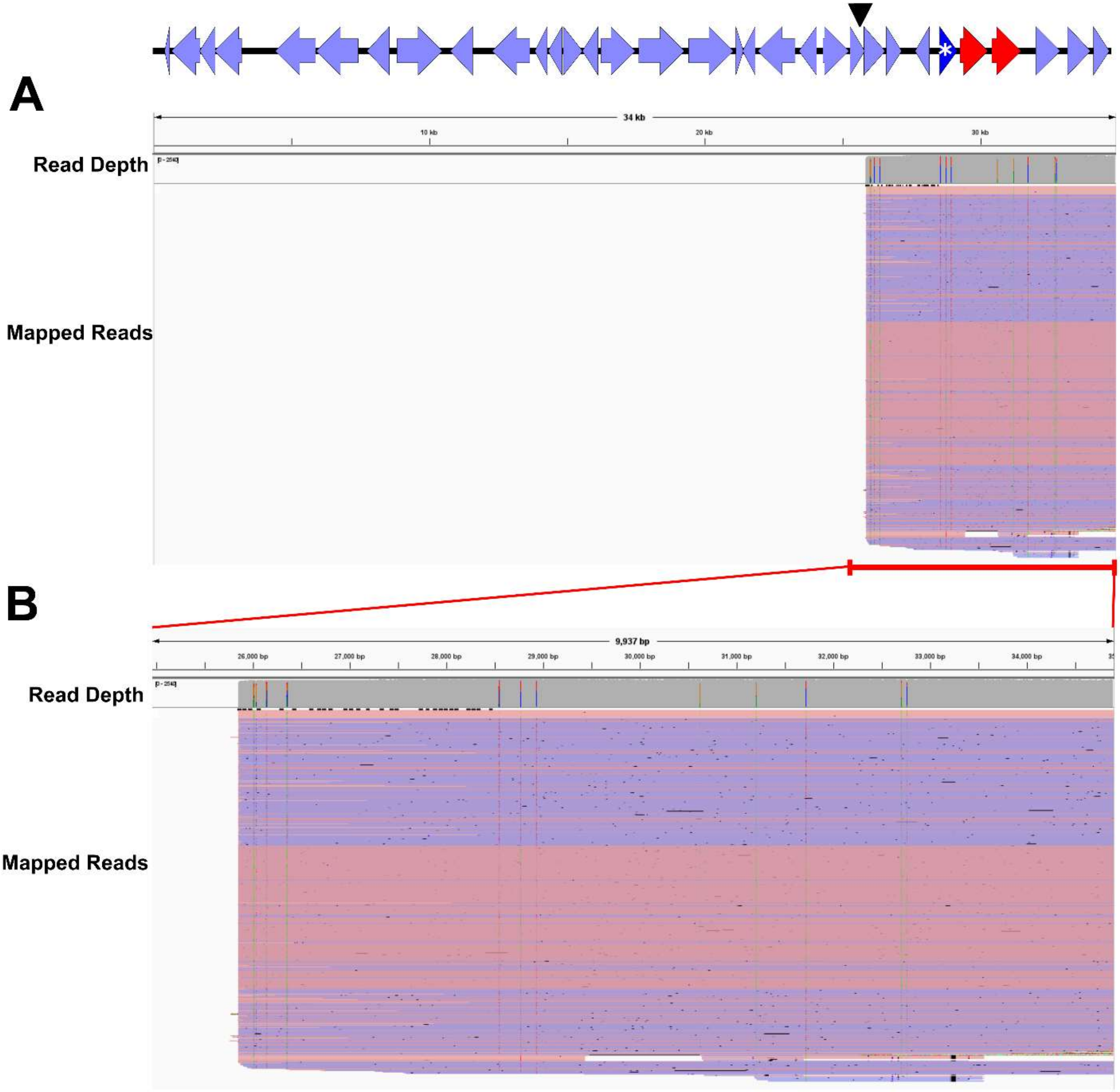
A
34 kb
10 kb
20 kb
30 kb
Read Depth
Mapped Reads
B
9,937 bp
26,000 bp
27,000 bp
28,000 bp
29,000 bp
30,000 bp
31,000 bp
32,000 bp
33,000 bp
34,000 bp
Read Depth
Mapped Reads

Figure S17. Mapped adaptive sampling reads for *B. venezuelensis* RMA01 lp35**.** Shown are the graphical representations of *B. venezuelensis* RMA01's lp35 plasmid and the BAM file visualization in the Integrated Genomics Viewer. In the graphical representation of the plasmids, the lavender arrows indicate ORFs and their directions, blue triangles indicate *vsp* alleles, and red indicates *vlp* alleles. The blue triangles with the asterisk indicate the *vmp* expression site. The black triangle represents the location of the BstXI restriction site on this plasmid. Only primary mapped reads are shown. Supplementary alignments have been linked with the forward alignment shown in red with the reverse alignment overlayed in blue. Where reads conflict in sequence with the reference, the conflicting base in the read is colored green for adenine, blue for cytosine, red for thymine, and yellow for guanine. Gaps or deletions are shown in the read as a black line. Insertions are indicated with a purple line in the read; however, indels <10bp have been masked. Reads mapping across the length of the plasmid are shown in **A.** Panel **B** is a enlarged view of the reads mapping to the 3' end.

Figure S18

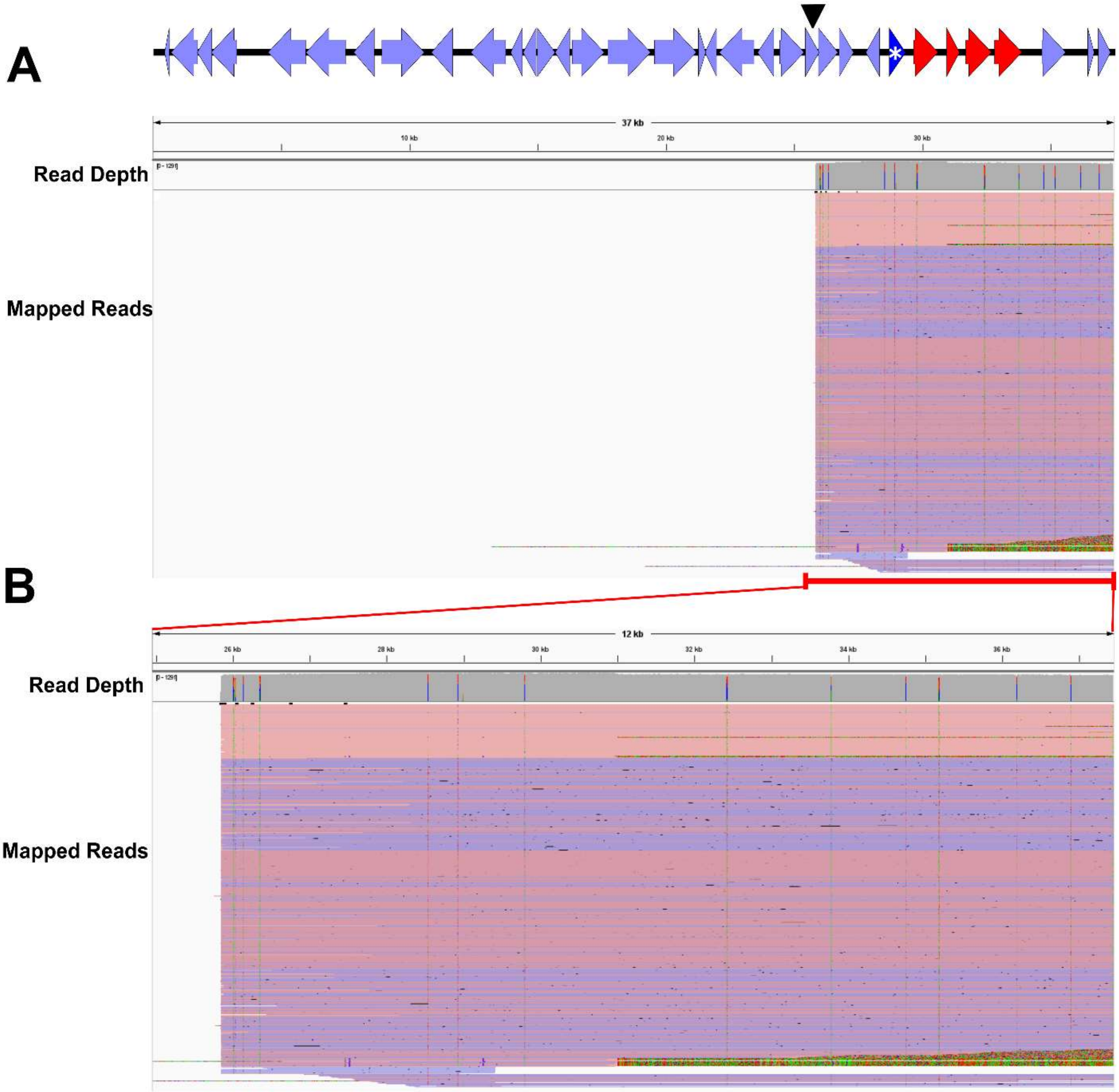
A
37 kb
10 kb
20 kb
30 kb
Read Depth
Mapped Reads
B
12 kb
26 kb
28 kb
30 kb
32 kb
34 kb
36 kb
Read Depth
Mapped Reads

Figure S18. Mapped adaptive sampling reads for *B. venezuelensis* RMA01 lp37**.** Shown are the graphical representations of *B. venezuelensis* RMA01's lp37 plasmid and the BAM file visualization in the Integrated Genomics Viewer. In the graphical representation of the plasmids, the lavender arrows indicate ORFs and their directions, blue triangles indicate *vsp* alleles, and red indicates *vlp* alleles. The blue triangles with the asterisk indicate the *vmp* expression site. The black triangle represents the location of the BstXI restriction site on this plasmid. Only primary mapped reads are shown. Supplementary alignments have been linked with the forward alignment shown in red with the reverse alignment overlayed in blue. Where reads conflict in sequence with the reference, the conflicting base in the read is colored green for adenine, blue for cytosine, red for thymine, and yellow for guanine. Gaps or deletions are shown in the read as a black line. Insertions are indicated with a purple line in the read; however, indels <10bp have been masked. Reads mapping across the length of the plasmid are shown in **A.** Panel **B** is a enlarged view of the reads mapping to the 3' end.

Figure S19

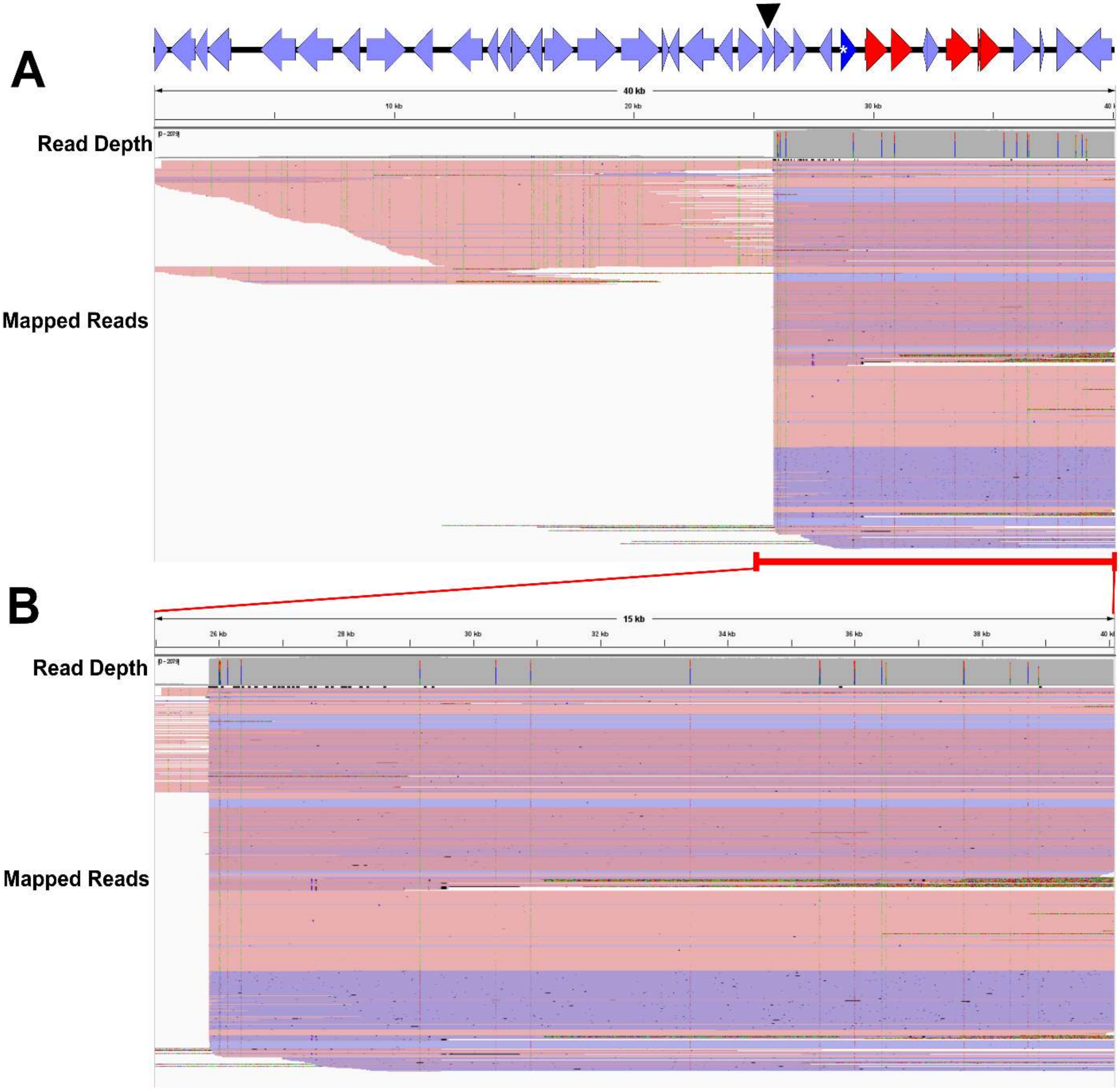
A
40 kb
10 kb
20 kb
30 kb
Read Depth
Mapped Reads
B
15 kb
26 kb
28 kb
30 kb
32 kb
34 kb
36 kb
38 kb
Read Depth
Mapped Reads

Figure S19. Mapped adaptive sampling reads for *B. turicatae* 91E135 lp40**.** Shown are the graphical representations of *B. turicatae* 91E135 lp40 plasmid and the BAM file visualization in the Integrated Genomics Viewer. In the graphical representation of the plasmids, the lavender arrows indicate ORFs and their directions, blue triangles indicate *vsp* alleles, and red indicates *vlp* alleles. The blue triangles with the asterisk indicate the *vmp* expression site. The black triangle represents the location of the BstXI restriction site on this plasmid. Only primary mapped reads are shown. Supplementary alignments have been linked with the forward alignment shown in red with the reverse alignment overlayed in blue. Where reads conflict in sequence with the reference, the conflicting base in the read is colored green for adenine, blue for cytosine, red for thymine, and yellow for guanine. Gaps or deletions are shown in the read as a black line. Insertions are indicated with a purple line in the read; however, indels <10bp have been masked. Reads mapping across the length of the plasmid are shown in **A.** Panel **B** is a enlarged view of the reads mapping to the 3' end.

Figure S20

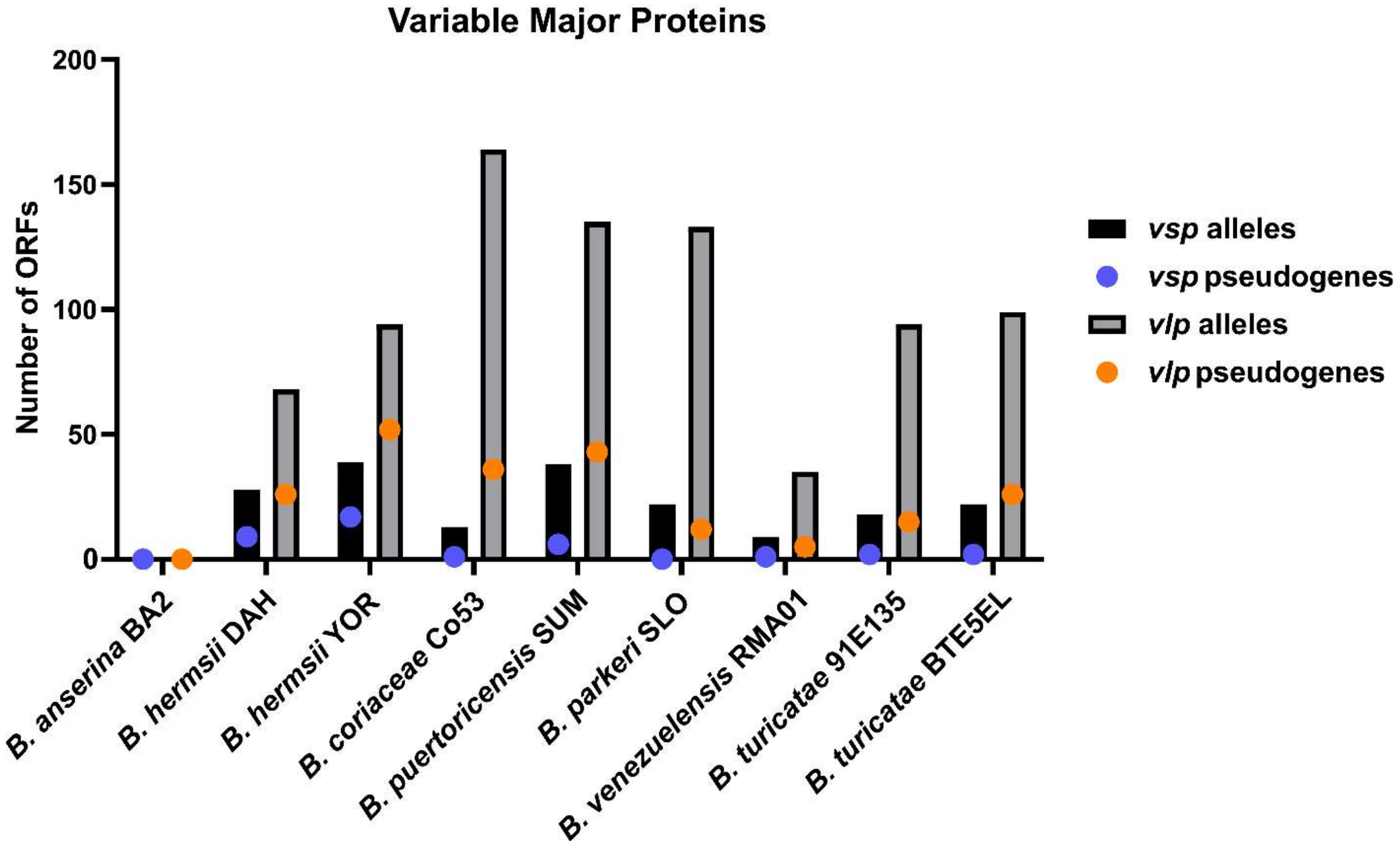

Figure S20. *vmp* allele composition. Total numbers of *vmp* alleles (including silent, archived genes as well as predicted pseudogenes) were determined from the InterProScan data for each isolate and are shown as bars. Pseudogenes were determined by PGAP annotation. The number of pseudogenes, as part of the whole *vmp* allele complement, are shown as circles. Note: *B. anserina* BA2 had only one vsp allele and no *vmp* pseudogenes.

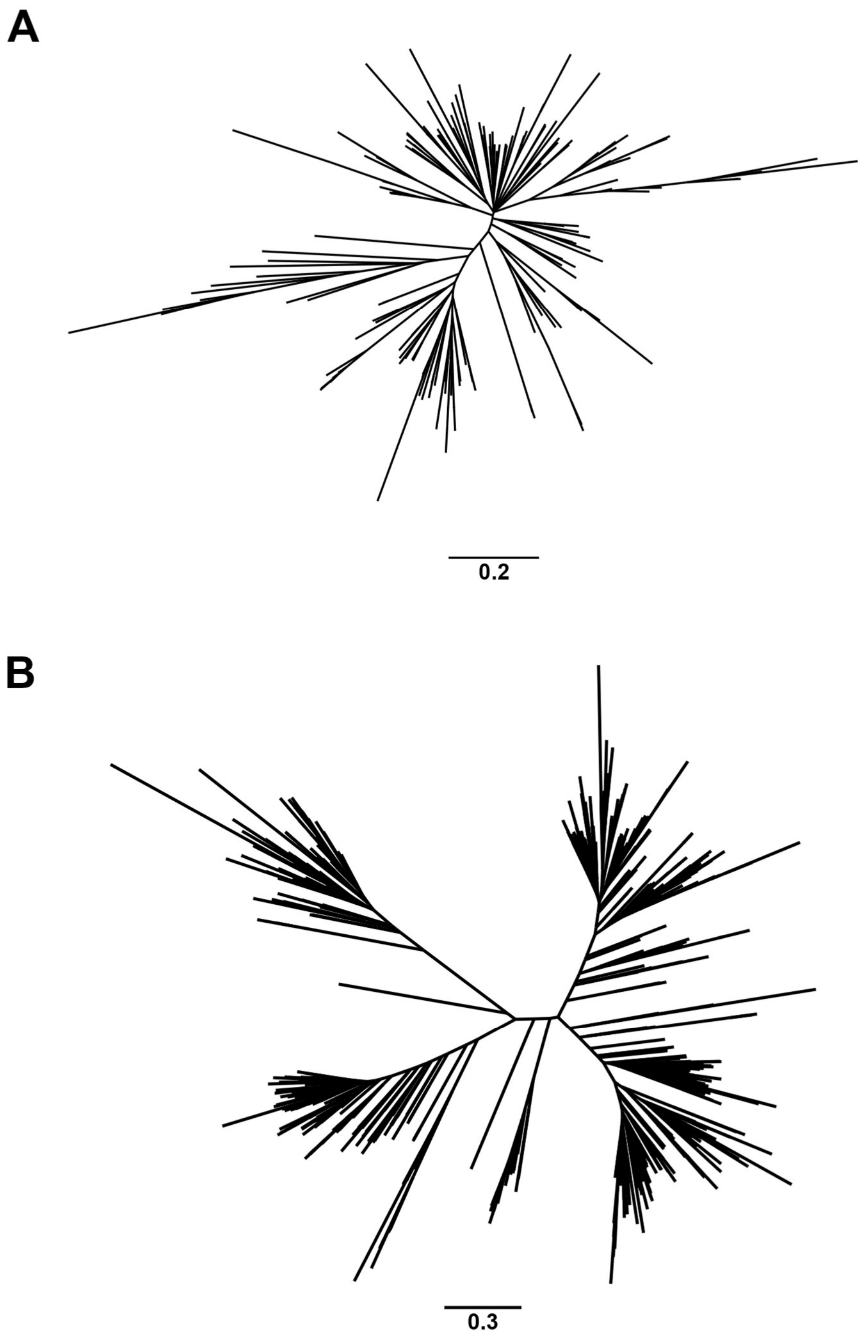

Figure S21

Figure S21. Combined phylogenetic analysis of *vmp* alleles. A maximum-likelihood tree was inferred with 1,000 ultrafast bootstrap replicates for all *vsp* (**A**) and *vlp* (**B**) alleles and pseudogenes. Branches with less than 50% support were collapsed. Unrooted trees were visualized in FigTree. The scale bar represents substitutions per site.

Figure S22

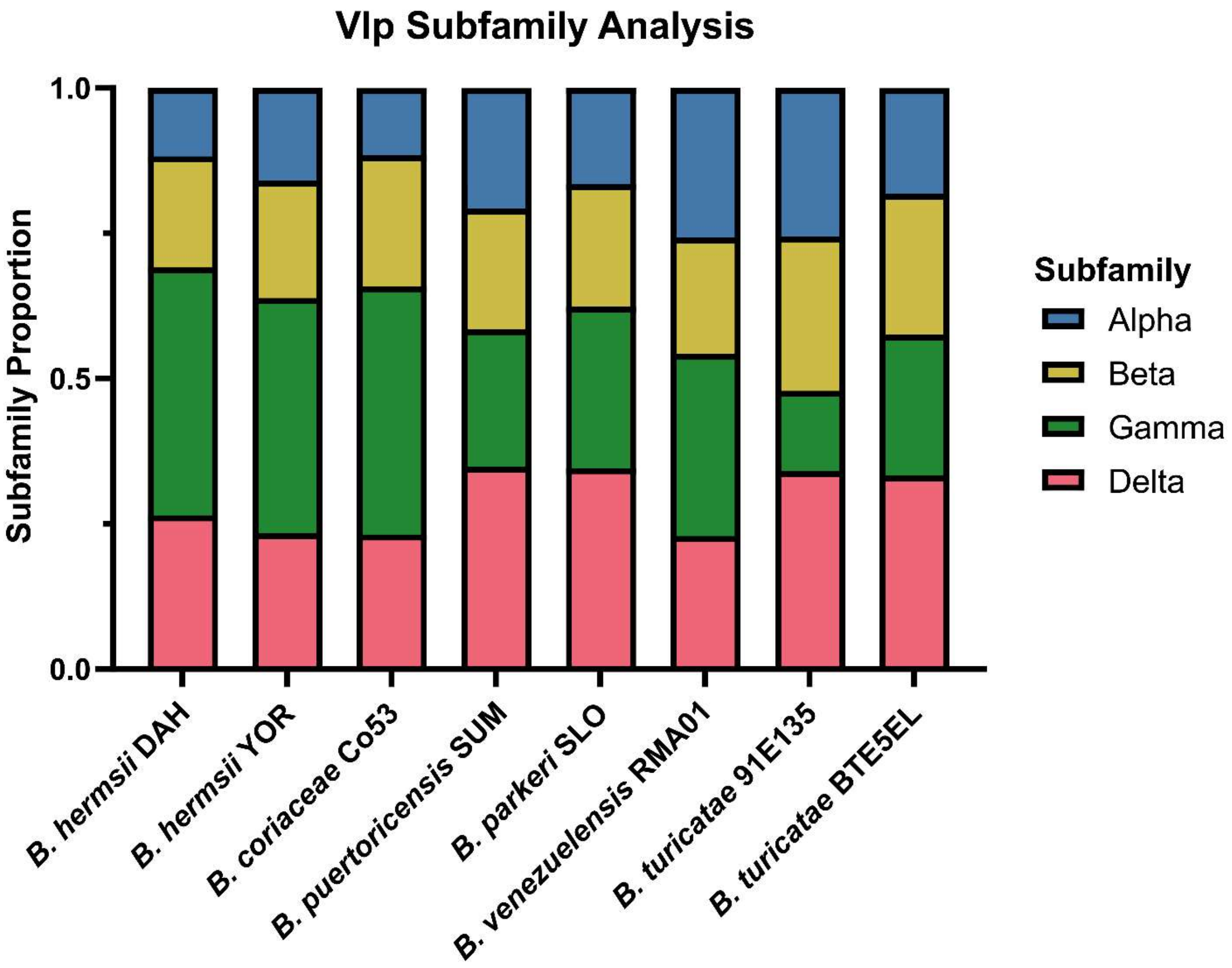

Figure S22. *vlp* subfamily proportions. The proportion that each *vlp* subfamily contributed to the whole *vlp* complement of a given genome was determined using the phylogenetic analysis from **Figure 6**.
